# Myxedema Coma Despite Mild Biochemical Hypothyroidism: A Case of Profound Clinical Decompensation

**DOI:** 10.7759/cureus.113595

**Published:** 2026-07-29

**Authors:** Sarra K Mohamed Ahmed, Dalia Salih, Razan K MOHAMEDAHMED, Kamil Ahmed

**Affiliations:** 1 Internal Medicine, Walsall manor hospital, Walsall, GBR; 2 Geriatrics, Walsall Manor Hospital, Walsall, GBR; 3 Internal Medicine, Walsall Manor Hospital, Walsall, SAU; 4 Internal Medicine, Al-Adwani General Hospital, Taif, SAU

**Keywords:** altered mental state, bradycardia, diagnostic challenge, endocrinology emergency, hypothermia, hypothyroidism, myxedema coma, popoveniuc score

## Abstract

Myxedema coma is a rare endocrine emergency with high mortality that is typically associated with severe biochemical hypothyroidism. However, profound clinical decompensation may occur despite only modest abnormalities in thyroid function tests, making diagnosis challenging. We report the case of an 87-year-old woman with long-standing hypothyroidism, primary adrenal insufficiency, pituitary pathology, and ischemic heart disease who presented with an *Escherichia coli* urinary tract infection and subsequently developed profound hypothermia, severe bradycardia, hypotension, reduced consciousness, and gastrointestinal hypomotility despite appropriate antimicrobial therapy. Thyroid function tests demonstrated only mildly elevated thyroid-stimulating hormone with near-normal free thyroxine concentrations. Repeat clinical assessment revealed delayed relaxation of the ankle reflexes. Retrospective application of the Popoveniuc diagnostic scoring system gave a score of 105, providing strong support for the diagnosis of myxedema coma. The end-of-life care pathway was reversed, and treatment with intravenous hydrocortisone, intravenous levothyroxine, active warming, and supportive care resulted in marked clinical recovery. This case highlights the importance of recognizing myxedema coma as a clinical diagnosis, maintaining a high index of suspicion despite relatively mild biochemical abnormalities, and undertaking repeated clinical reassessments in elderly patients with impaired endocrine reserve.

## Introduction

Myxedema coma is a rare endocrine emergency and the most severe presentation of hypothyroidism. Mortality rates reported range from 30% to 50% despite advances in intensive care and endocrine management [1,2]. It is seen predominantly in elderly women with chronic hypothyroidism and is often precipitated by physiological stressors such as infection, cardiovascular events, cold exposure, trauma, or sedative medications [1,2]. Although myxedema coma is a widely used term, many patients present with progressive confusion, lethargy, or reduced consciousness rather than frank coma [1].

This occurs when the adaptive physiological mechanisms that maintain homeostasis in chronic hypothyroidism are overwhelmed [1]. Patients compensate with chronic peripheral vasoconstriction, increased systemic vascular resistance, and decreased circulating blood volume to preserve core temperature and maintain the perfusion of vital organs [1]. When exposed to an acute physiological stressor, these compensatory mechanisms can become exhausted [1]. Acute physiological stressors can exhaust these compensatory mechanisms, leading to impaired thermoregulation, cardiovascular collapse, respiratory insufficiency, and neurological deterioration [1]. Thyroid hormone levels in the serum do not always reflect the severity of clinical disease, as the pathology is a deficiency of intracellular triiodothyronine (T3) leading to impaired mitochondrial oxidative metabolism, reduced oxygen consumption, diminished ATP production, and multisystem organ dysfunction [1]. Therefore, life-threatening clinical manifestations may occur despite mild abnormalities in thyroid function tests, particularly in patients with compromised endocrine reserve due to pituitary disease, adrenal insufficiency, or co-existing critical illness [1,3,4].

Diagnosis remains challenging as myxedema coma can mimic more common conditions, including sepsis, metabolic encephalopathy, adrenal crisis, and decompensated cardiac disease [1,4]. Clinical scoring systems encompass hypothermia, altered mental status, cardiovascular dysfunction, metabolic abnormalities, gastrointestinal involvement, and precipitating events [5]. The most widely used scoring system is the Popoveniuc diagnostic scoring system [5]. This tool aids in the diagnosis when the biochemical findings are disproportionately mild.

Management includes initiating thyroid hormone replacement, empirical glucocorticoid therapy until adrenal insufficiency is excluded or adequately treated, aggressive supportive care, and treatment of the precipitating illness [1,6]. Early recognition remains crucial because clinical improvement often precedes full biochemical normalization [4].

We report an unusual case of profound myxedema coma in an elderly woman with long-standing hypothyroidism, primary adrenal insufficiency, pituitary pathology, and an acute *Escherichia coli* urinary tract infection, who developed severe cardiovascular and neurologic collapse despite relatively modest abnormalities in thyroid function tests. This case highlights the importance of recognizing reduced endocrine reserve, maintaining a high index of suspicion despite apparently reassuring biochemical findings, and repeating clinical reassessment when patients continue to deteriorate despite appropriate treatment of the presumed underlying diagnosis.

## Case presentation

An 87-year-old woman presented with fever, confusion, reduced mobility, and progressive functional decline. Her medical history included longstanding primary hypothyroidism treated with levothyroxine 125 µg once daily, with satisfactory adherence confirmed by both the patient and her family; primary adrenal insufficiency diagnosed previously by a short Synacthen test and managed with maintenance hydrocortisone replacement (10 mg on waking, 5 mg at midday, and 5 mg in the evening); pituitary pathology under endocrine follow-up; and ischemic heart disease. She had previous hospital admissions with recurrent urinary tract infections, hypothermia, and electrolyte disturbances, during which temporary escalations of glucocorticoid replacement according to sick-day rules had been recommended.

She was admitted with sepsis secondary to urinary tract infection due to *E. coli*, which was later confirmed on urine culture. Blood cultures were negative. Intravenous antibiotic therapy was started. The admission procalcitonin was 5.0 ng/mL, consistent with bacterial infection. During treatment, inflammatory markers improved progressively, with procalcitonin decreasing to 1.5 ng/mL, suggesting an appropriate response to antimicrobial therapy.

Her clinical picture progressively deteriorated despite treatment of the underlying infection. On day four of her hospital stay, she developed profound hypothermia with a core temperature of 33°C, severe sinus bradycardia (30-35 beats/minute), hypotension (75/40 mmHg), and marked reduction in consciousness that necessitated urgent review by the medical emergency team. Intravenous fluids and atropine moderately improved her hemodynamics, raising her heart rate with intravenous fluids and atropine, with heart rate increasing to 35-40 beats/minute and blood pressure to 85/50 mmHg. Overnight, an end-of-life care pathway was initiated due to her ongoing decline, advanced age, and multiple comorbidities.

During the morning post-take ward round on hospital day five, the daytime medical team undertook a comprehensive clinical reassessment. Her medical history was reviewed and revealed a history of long-standing hypothyroidism, primary adrenal insufficiency, and pituitary pathology. Physical examination showed persistent hypothermia, severe bradycardia, hypotension, and profoundly decreased responsiveness with a Glasgow Coma Scale score of 9/15. Neurological examination demonstrated delayed relaxation of the ankle reflexes, which are classical features of severe hypothyroidism. Abdominal examination revealed a soft, non-tender abdomen with sluggish bowel sounds, suggestive of gastrointestinal hypomotility. The combination of these findings raised a strong clinical suspicion of myxedema coma, despite only relatively modest abnormalities on thyroid function tests.

**Figure 1:**
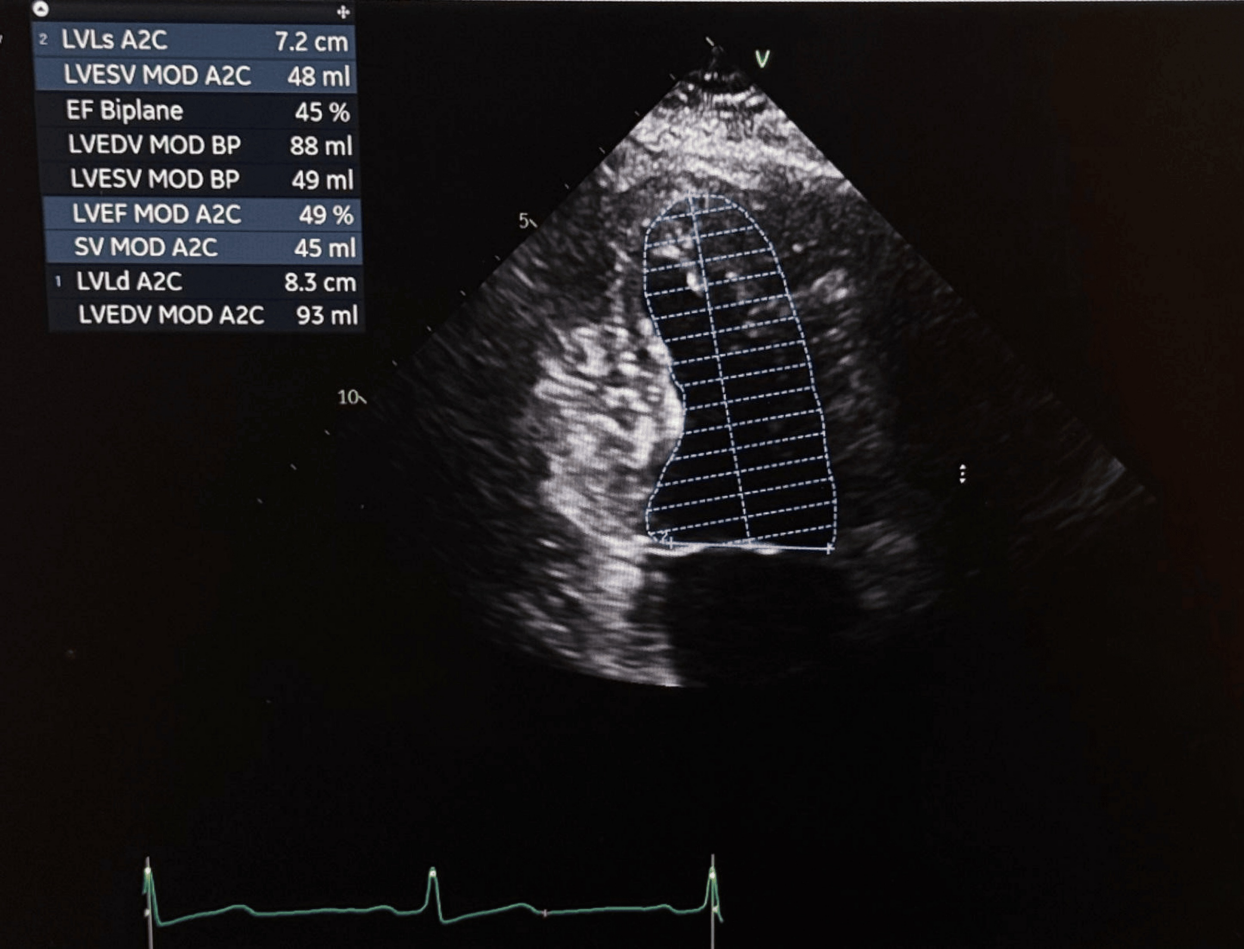
Transthoracic echocardiography demonstrating mildly impaired left ventricular systolic function with a biplane left ventricular ejection fraction of 45% calculated using Simpson’s biplane method.

Initial biochemical investigations showed a thyroid-stimulating hormone (TSH) concentration of 14.8 mIU/L and a free thyroxine (FT4) concentration of 12.3 pmol/L. Further investigations revealed hemoglobin of 76 g/L, platelet count of 120 × 10⁹/L, albumin of 24 g/L, estimated glomerular filtration rate of 35 mL/minute/1.73 m², and brain natriuretic peptide (BNP) of 6,660 pg/mL. Due to the severe hemodynamic instability and raised BNP, a transthoracic echocardiogram was performed. It showed a non-dilated left ventricle with concentric remodeling, mild left ventricular systolic dysfunction with a biplane ejection fraction of 45%, and impaired diastolic function with normal filling pressures. These results were considered insufficient to explain the severity of the patient’s cardiovascular collapse and were consistent with her known ischemic heart disease, thereby supporting an alternative diagnosis (Figure 1).

The differential diagnosis included persistent sepsis, adrenal crisis, cardiogenic shock, metabolic encephalopathy, and myxedema coma. Given the neurological and cardiovascular deterioration despite appropriate antimicrobial therapy, the improvement in inflammatory markers made persistent uncontrolled sepsis less likely. Adrenal insufficiency was considered an important contributory factor but could not account for the profound hypothermia, delayed relaxation of the ankle reflexes, and the characteristic cardiovascular manifestations. The Popoveniuc diagnostic scoring system applied retrospectively gave a score of 105, which strongly supported the clinical diagnosis (Table 1).

**Table 1:** Retrospective application of the Popoveniuc diagnostic scoring system. A score ≥60 is highly suggestive/diagnostic of myxedema coma.

Diagnostic variable	Finding	Score
Thermoregulatory dysfunction	Temperature: 33°C	20
Central nervous system	Markedly reduced consciousness	20
Gastrointestinal dysfunction	Sluggish bowel sounds/ileus	15
Cardiovascular dysfunction	Heart rate: 30 beats/minute	30
Cardiovascular dysfunction	Hypotension (75/40 mmHg)	20
Precipitating event	*Escherichia coli* urinary tract infection	10
Total score	105	

Given the high clinical suspicion of a reversible endocrine emergency, the end-of-life care pathway was reversed immediately during the morning ward round on hospital day five, and active treatment was commenced. Hydrocortisone 100 mg intravenously as a stat dose followed by 50 mg intravenously four times a day was given. A loading dose of 200 µg (4 µg/kg) of levothyroxine was given intravenously, followed by 75 µg intravenously once daily. Simultaneous active external rewarming, continued antimicrobial therapy, and supportive care were given.

The patient then demonstrated progressive clinical improvement. Her consciousness slowly returned to the baseline level; her body temperature, heart rate, and blood pressure normalized; and her overall functional status improved. Serial abdominal examinations showed a return of gastrointestinal function, with bowel sounds returning to normal on day two after endocrine replacement and spontaneous bowel opening the following day. When bowel function had returned and oral absorption was thought to be reliable, intravenous levothyroxine was stopped, and her usual oral levothyroxine dose of 125 µg once daily was restarted.

Serial thyroid function tests, as expected, reflected the biochemical response to treatment. Forty-eight hours after the start of endocrine replacement, the TSH was 14.0 mIU/L, and the FT4 was 25.0 pmol/L. One week later, repeat testing showed a gradual fall in TSH to 12.0 mIU/L with FT4 at 20.4 pmol/L, consistent with progressive biochemical recovery. The sequence of clinical events is summarized in Table 2. The major clinical and laboratory findings are presented in Table 3.

**Table 2:** Timeline of clinical events.

Hospital day	Clinical events
Day 1	Admission with fever, confusion, reduced mobility, and Escherichia coli urinary tract infection. Intravenous antibiotics commenced
Days 2–4	Clinical deterioration despite improving inflammatory markers
Night of day 4	Core temperature: 33°C, heart rate: 30–35 beats/minute, blood pressure: 75/40 mmHg, reduced consciousness. Medical Emergency Team review. End-of-life care pathway initiated
Morning of day 5	Clinical reassessment identified delayed relaxation of the ankle reflexes, severe hypothermia, bradycardia, and hypotension. High suspicion of myxoedema coma. End-of-life care pathway reversed
Day 5	Intravenous hydrocortisone 100 mg stat followed by 50 mg intravenously four times daily; intravenous levothyroxine loading dose 200 µg followed by 75 µg intravenously daily; active warming and supportive care commenced
Day 7	Bowel sounds returned to normal
Day 8	Spontaneous bowel opening. Intravenous levothyroxine changed to oral levothyroxine 125 µg daily
Following days	Progressive neurological, cardiovascular, gastrointestinal, and biochemical recovery

**Table 3:** Clinical and laboratory findings during clinical deterioration and response to treatment. ^†^: The patient’s consciousness progressively returned to baseline following endocrine replacement therapy. ^‡^: Measured after initiation of antimicrobial therapy, demonstrating improvement in the inflammatory response. TSH = thyroid-stimulating hormone; FT4 = free thyroxine; ALP = alkaline phosphatase; ALT = alanine aminotransferase; eGFR = estimated glomerular filtration rate; BNP = brain natriuretic peptide; LV = left ventricular; LVEF = left ventricular ejection fraction

Investigation	During clinical deterioration (hospital day 5)	48 hours after treatment	1 week after treatment	Reference range
Core temperature	33°C	Normalised	Normal	36.5–37.5°C
Glasgow Coma Scale	9/15	Improved 13/15	15/15 (baseline )^†^	15/15
Blood pressure	75/40 mmHg	85/50 mmHg	Normalized	—
Heart rate	30–35 beats/minute	35–40 beats/minute	Normalized	60–100 beats/minute
TSH	14.8 mIU/L	14.0 mIU/L	12.0 mIU/L	0.35–4.94 mIU/L
Free T4	12.3 pmol/L	25.0 pmol/L	20.4 pmol/L	9.0–19.0 pmol/L
Sodium	135 mmol/L	140 mmol/L	141 mmol/L	135–145 mmol/L
Potassium	3.5 mmol/L	3.8 mmol /L	4 mmol/L	3.5–5.3 mmol/L
Albumin	24 g/L	26 g/L	31 g/L	35–50 g/L
Total bilirubin	4 µmol/L	—	16 µmol/L	0–21 µmol/L
ALP	123 U/L	—	120 U/L	30–130 U/L
ALT	40 U/L	—	36 U/L	7–56 U/L
Hemoglobin	76 g/L	80 g/L	—	120–160 g/L
White blood cell count	4.20 × 10⁹/L	6 × 10⁹/L	—	4.0–11.0 × 10⁹/L
Platelets	120 × 10⁹/L	139 × 10⁹/L	—	150–400 × 10⁹/L
eGFR	35 mL/minute/1.73 m²	40 mL/minute/1.73 m²	55 mL/minute/1.73 m²	>90 mL/minute/1.73 m²
Procalcitonin	1.5 ng/mL^‡^	0.48 ng/mL	0.01 ng/mL	<0.05 ng/mL
Urine culture	Escherichia coli growth	—	—	No growth
Blood cultures	Negative	—	—	Negative
BNP	6,660 pg/mL	—	—	—

## Discussion

Myxoedema coma represents the most severe manifestation of hypothyroidism and remains a rare endocrine emergency associated with high mortality despite advances in intensive care and endocrine management [1,2]. Although myxedema coma is classically associated with profound biochemical hypothyroidism, this case demonstrates that severe clinical decompensation may occur despite only relatively modest abnormalities in the thyroid function tests. Our patient presented with profound hypothermia, severe bradycardia, hypotension, altered mental status, and gastrointestinal hypomotility [1,4]. Thyroid function tests indicated that the TSH level was only slightly higher than normal and that the FT4 level was within the normal range. This emphasizes the fact that myxedema coma remains largely a clinical diagnosis, with biochemical investigations being more supportive than definitive [1,4].

The apparent discrepancy between the severity of clinical illness and relatively modest biochemical abnormalities is biologically plausible. The life-threatening manifestations of myxedema coma are primarily due to inadequate intracellular T3 activity rather than circulating thyroid hormone concentrations alone. Reduced intracellular T3 impairs mitochondrial oxidative metabolism, decreases oxygen consumption, and decreases ATP production, leading to multisystem organ dysfunction [1]. In patients with longstanding hypothyroidism, physiological homeostasis is achieved through adaptive neurovascular mechanisms, which include chronic peripheral vasoconstriction, increased systemic vascular resistance, and decreased circulating blood volume. These compensatory mechanisms can be overwhelmed by acute physiologic stressors such as infection, leading to cardiovascular collapse, neurologic deterioration, impaired thermoregulation, and respiratory compromise in the presence of apparently modest biochemical abnormalities. This pathophysiological framework explains why routine thyroid function tests may not accurately reflect the severity of tissue hypothyroidism [1,5].

The concept of reduced endocrine reserve is particularly relevant in this case. Endocrine reserve is the ability of endocrine organs to preserve physiological homeostasis in the face of acute stress [1,6]. Our patient had longstanding hypothyroidism, primary adrenal insufficiency, and pituitary pathology, all of which likely impaired her ability to compensate during acute illness. The documented *E. coli* urinary tract infection was, therefore, probably the precipitant, but not the sole etiology of her decline [2]. In addition, pituitary pathology may have affected the interpretation of thyroid function tests so that circulating hormone concentrations were less representative of thyroid hormone activity in tissues. Crucially, the patient had been compliant with long-term levothyroxine replacement before admission, making poor treatment adherence an unlikely explanation for her presentation. This case, however, supports earlier reports that severe tissue hypothyroidism may occur when endocrine reserve becomes critically impaired despite apparently adequate replacement therapy [5,7,8]. Our patient also had anemia (hemoglobin 76g/L) and mild thrombocytopenia. Hematological abnormalities have been described in severe hypothyroidism and are thought to result from reduced bone marrow activity and impaired hematopoiesis secondary to thyroid hormone deficiency. In this patient, however, these abnormalities were likely multifactorial, reflecting acute infection, chronic comorbidities, and severe endocrine decompensation rather than myxedema coma alone [9].

The differential diagnosis was challenging because there were several other possible explanations [4]. The patient’s decline was thought to be secondary to ongoing sepsis, adrenal crisis, cardiogenic shock, and metabolic encephalopathy. The urinary tract infection certainly contributed to the initial presentation, but persistent uncontrolled sepsis became progressively less likely as inflammatory markers improved following appropriate antimicrobial therapy, while neurological and cardiovascular deterioration continued. Although adrenal insufficiency probably contributed to the overall clinical picture, it was insufficient to account for the profound hypothermia, delayed relaxation of the ankle reflexes, and characteristic cardiovascular manifestations of severe hypothyroidism. Transthoracic echocardiography showed only mildly impaired left ventricular systolic function, which was not sufficient to explain the severity of hemodynamic collapse. The Popoveniuc scoring system for diagnosis was applied retrospectively and resulted in a score of 105, which strongly supported the clinical diagnosis of myxedema coma [4].

Perhaps the most important learning point of this case is the value of repeated clinical reassessment. The patient’s poor prognosis resulted in the initiation of an end-of-life care pathway during overnight deterioration. However, the day team, on further assessment at the post-take ward round, including a detailed review of the patient’s endocrine history and a targeted physical examination, noted delayed relaxation of the ankle reflexes as well as profound hypothermia, severe bradycardia, hypotension, and altered mental status. The classic clinical features were recognized, and the end-of-life care pathway was reversed immediately, and treatment for a reversible endocrine emergency was initiated. This case, therefore, emphasizes the importance of maintaining diagnostic vigilance and reconsidering the diagnosis when patients continue to deteriorate despite apparently appropriate treatment of the presumed underlying condition.

Management of myxedema coma includes prompt thyroid hormone replacement, empirical glucocorticoid therapy until adrenal insufficiency has been excluded or adequately treated, aggressive supportive care, and treatment of the precipitating illness [1,6]. Our patient was treated with intravenous hydrocortisone 100 mg stat, followed by 50 mg intravenously four times daily. She was also given an intravenous levothyroxine loading dose of 200 µg (4 µg/kg) and maintenance intravenous levothyroxine of 75 µg once daily [6]. A conservative dose of loading levothyroxine was chosen in view of the patient’s advanced age, low body weight, and underlying ischemic heart disease and was a compromise between the need for rapid thyroid hormone replacement and the risk of precipitating myocardial ischemia or arrhythmias. Gastrointestinal hypomotility is common in severe hypothyroidism and may interfere with enteral drug absorption [1]. Therefore, intravenous levothyroxine was maintained until bowel function was re-established. Bowel sounds were recovered, and she was opening her bowels spontaneously before her usual oral levothyroxine dose was resumed, providing objective evidence of reliable oral absorption.

In our patient, the biochemical response was in line with the expected physiological response to treatment [1,4]. FT4 concentrations increased rapidly after intravenous levothyroxine administration, whereas TSH decreased only slowly over the following week. This pattern emphasizes an important clinical principle: successful treatment of myxedema coma should be judged primarily on clinical improvement rather than on rapid biochemical normalization, as restoration of pituitary feedback and suppression of TSH occurs over several days to weeks.

Several published case reports have described myxedema coma occurring in patients with subclinical hypothyroidism or relatively mild thyroid function test abnormalities, emphasizing that biochemical findings alone should not delay diagnosis or treatment. These reports and the present case reinforce the importance of careful clinical assessment when the overall presentation is suggestive of severe hypothyroidism despite apparently reassuring laboratory findings [5,7,10-12].

This report does have some limitations. The diagnosis was made retrospectively based on the clinical picture, the exclusion of other diagnoses, the retrospective application of the Popoveniuc diagnostic scoring system, and the subsequent response of the patient to treatment. Moreover, the infection, glucocorticoid replacement, thyroid hormone replacement, active warming, and supportive measures all contributed to the patient’s recovery, and thus it is not possible to ascertain the exact contribution of each individual measure. But the continued clinical deterioration despite normalization of inflammatory markers, coupled with the rapid clinical improvement after endocrine replacement, makes severe endocrine decompensation the most likely cause of the patient’s presentation.

## Conclusions

Myxedema coma remains a rare but life-threatening endocrine emergency in which the severity of clinical illness may be disproportionate to thyroid function test abnormalities. This case illustrates that the diagnosis should not be ruled out based on relatively modest biochemical findings when classical clinical features of hypothermia, severe bradycardia, hypotension, altered mental status, and delayed relaxation of the ankle reflexes are present. Patients with impaired endocrine reserve and concomitant endocrine disorders may be especially vulnerable to profound endocrine decompensation secondary to an acute physiologic stressor. This case also illustrates the importance of repeated clinical reassessment when patients continue to deteriorate despite apparently appropriate treatment of the presumed underlying diagnosis. Identification of a reversible endocrine emergency during the post-take ward round resulted in the timely cessation of the end-of-life care pathway and initiation of appropriate endocrine replacement therapy. Although the Popoveniuc diagnostic scoring system supported the diagnosis in retrospect and the clinical response was consistent, this case reinforces that myxedema coma is still a clinical diagnosis requiring a high index of suspicion and timely treatment to improve outcomes.
